# Impaired Pain Processing at a Brainstem Level Is Involved in Maladaptive Neuroplasticity in Patients with Chronic Complex Regional Pain Syndrome

**DOI:** 10.3390/ijms232315368

**Published:** 2022-12-06

**Authors:** Pauline Thoma, Nina Drämel, Matthias Grothe, Martin Lotze, Robert Fleischmann, Sebastian Strauss

**Affiliations:** 1 Department of Neurology, University Medicine Greifswald, 17475 Greifswald, Germany; 2 Functional Imaging Unit, Center for Diagnostic Radiology, University Medicine Greifswald, 17475 Greifswald, Germany

**Keywords:** complex regional pain syndrome, CRPS, central sensitization, brainstem, blink reflex

## Abstract

Neuroinflammatory mechanisms and maladaptive neuroplasticity underlie the progression of complex regional pain syndrome (CRPS), which is prototypical of central neuropathic pain conditions. While cortical maladaptive alterations are well described, little is known about the contribution of the brainstem to the pathophysiology. This study investigates the role of pain-modulatory brainstem pathways in CRPS using the nociceptive blink reflex (nBR), which not only provides a direct read-out of brainstem excitability and habituation to painful stimuli but may also be suitable for use as a diagnostic biomarker for CRPS. Thirteen patients with CRPS and thirteen healthy controls (HCs) participated in this prospective case-control study investigating the polysynaptic trigemino-cervical (R2) nBR response. The R2 area and its habituation were assessed following repeated supraorbital electrical stimulation. Between-group comparisons included evaluations of diagnostic characteristics as a potential biomarker for the disease. Patients with CRPS showed a substantial decrease in habituation on the stimulated (Cohen’s d: 1.3; *p* = 0.012) and the non-stimulated side (Cohen’s d: 1.1; *p* = 0.04). This is the first study to reveal altered nBR habituation as a pathophysiological mechanism and potential diagnostic biomarker in CRPS. We confirmed previous findings of altered nBR excitability, but the diagnostic accuracy was inferior. Future studies should investigate the nBR as a marker of progression to central mechanisms in CRPS and as a biomarker to predict treatment response or prognosis.

## 1. Introduction

Complex regional pain syndrome (CRPS) is a chronic pain condition that follows limb trauma in about 7% of cases [1] and is accompanied by sensorimotor dysfunction [2]. Pathophysiological local inflammatory processes, nociceptive (peripheral) sensitization, vasomotor dysfunction, and maladaptive neuroplasticity are thought to account for the majority of clinical features of CRPS [3]. Maladaptive neuroplasticity involves mechanisms of central sensitization, which is thought to be a key pathophysiological mechanism of disease progression in CRPS [4]. A study investigating rodents demonstrated that mechanisms of central sensitization involve disinhibition at different levels of the nociceptive system, including spinal and trigeminal nociceptive neurons and thalamocortical nociceptive networks [5]. These changes promote alterations in different brain areas involved in sensorimotor function as well as emotional aspects of pain (amygdala, anterior cingulate cortex, and prefrontal cortex), which have been reported frequently in animal models and humans with CRPS [6,7].

**Figure 1:**
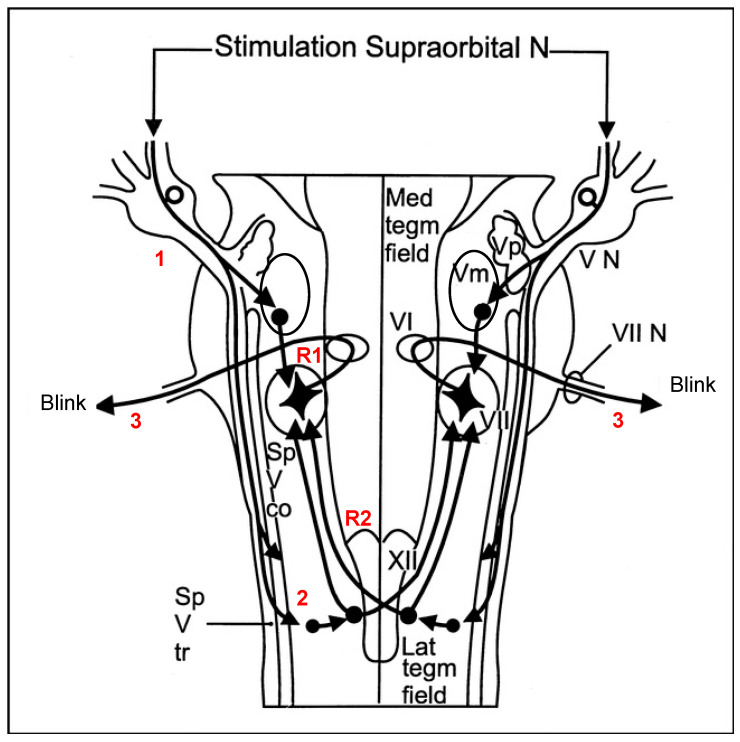
Schematic representation of the blink reflex pathways. The stimulus is perceived by the ipsilateral supraorbital nerve (1) and is conducted to the trigeminal motor nucleus (Vm), where the ipsilateral early response (R1) is obtained via an oligosynaptic pathway to the facial nucleus. The nociceptive stimulus is also conducted from the supraorbital nerve to the lower brainstem, including the caudal spinal trigeminal nuclei (2), via a long descending spinal tract of the trigeminal nerves (Sp V tr). After multiple interconnections, an ascending efferent impulse is then conducted bilaterally to VII (3) and consequently results in a bilateral late electrophysiological response (R2) and a visible bilateral eyeblink. Sp V co, spinal trigeminal complex; VI, abducens nucleus; VII N, facial nerve; VN, trigeminal sensory root; XII, hypoglossal nucleus; Lat tegm field, lateral tegmental field; Med tegm field, medial tegmental field. Modified from [15] and used with permission from the author.

While cortical secondary maladaptive alterations in CRPS are well established, little is known about the contribution of brainstem circuitry to the pathophysiology of CRPS. Brainstem pathways are assumed to be substantially involved in the endogenous pain-modulation network; they are therefore critical for the initiation and/or maintenance of pain and are thus predisposed to the development of chronic pain [8,9]. Brainstem pathways can be assessed neurophysiologically using the nociceptive blink reflex (nBR) [10]. The nBR consists of an early oligosynaptic ipsilateral R1 component and two late bilateral polysynaptic R2 (R2) [11]. The latter is thought to be mediated via nociceptive afferences of the trigeminal nerve throughout the tractus spinalis trigeminalis and second-order brain stem neurons [12,13,14,15] (for more details on brainstem pathways, see Figure 1). Methodologically, the R2 component can be quantified by the mean amplitude/area, which is understood to reflect excitability. The habituation of repeated stimulation is a marker of sensitization, which indicates a lack of inhibitory response [16,17]. Specifically, a lack of habituation has been identified as a key pathophysiological mechanism in different conditions such as migraines [18] and persistent idiopathic facial pain [19]. The clinical significance of the nBR is highlighted by its association with successful treatment responses and restored brainstem habituation deficits in patients with migraines [10].

In patients with CRPS, there is only preliminary evidence for altered brainstem excitability [20]. Understanding the role of habituation deficits at the brainstem level would improve the pathophysiological model of CRPS and thus help identify novel therapeutical targets (e.g., through non-invasive brain stimulation) and potential biomarkers for CRPS [21]. Both aspects are of substantial clinical interest since CRPS often has a poor prognosis despite treatment. It is known that fast initiation of treatment is critical to limit disease progression and improve patients’ quality of life. Therefore, early and correct diagnosis of CRPS is essential but often difficult. This study investigates the nBR to clarify the pathophysiology of disease progression in CRPS. We will furthermore explore the utility of the nBR as a biomarker for the diagnosis of CRPS.

The present study aims to fill the gap and examines several hypotheses, indicating an emphasized role of the brainstem in CRPS and its potential as a diagnostic biomarker through a more comprehensive assessment of the nBR. We hypothesized that (1) patients suffering from CRPS would show a substantial decrease in nBR as a potential diagnostic biomarker for mechanisms of central sensitization. In more detail, we expected a decrease in the habituation of the R2 component and an increase in the R2 area after repetitive stimulation. In addition, we expected (2) significant associations of clinical parameters (current pain levels, CRPS severity, and CRPS duration) and electrophysiological results (habituation and area of the R2 component, as specified in the German Clinical Trials Register (DRKSDRKS0002661).

## 2. Results

### 2.1. Demographics and Clinical Data

We enrolled 13 patients diagnosed with CRPS in our final analysis (9 females, aged 53.8 ± (SD) 17.8 years; 5 patients were left-handed; in 8 patients, the dominant hand was affected; time after disease onset: 78 ± 40 months).

The mean pain intensity levels in the resting and moving hands were 4.8 ± 1.9 and 7.3 ± 2, respectively. The CSS score (range: 7–15, median: 11) demonstrates that all patients showed severe CRPS symptoms. For an overview of the participants’ clinical, demographic, and testing data, see Table 1.

### 2.2. Blink Reflex Assessment—Differences between Patients with CRPS and Controls

**Figure 2:**
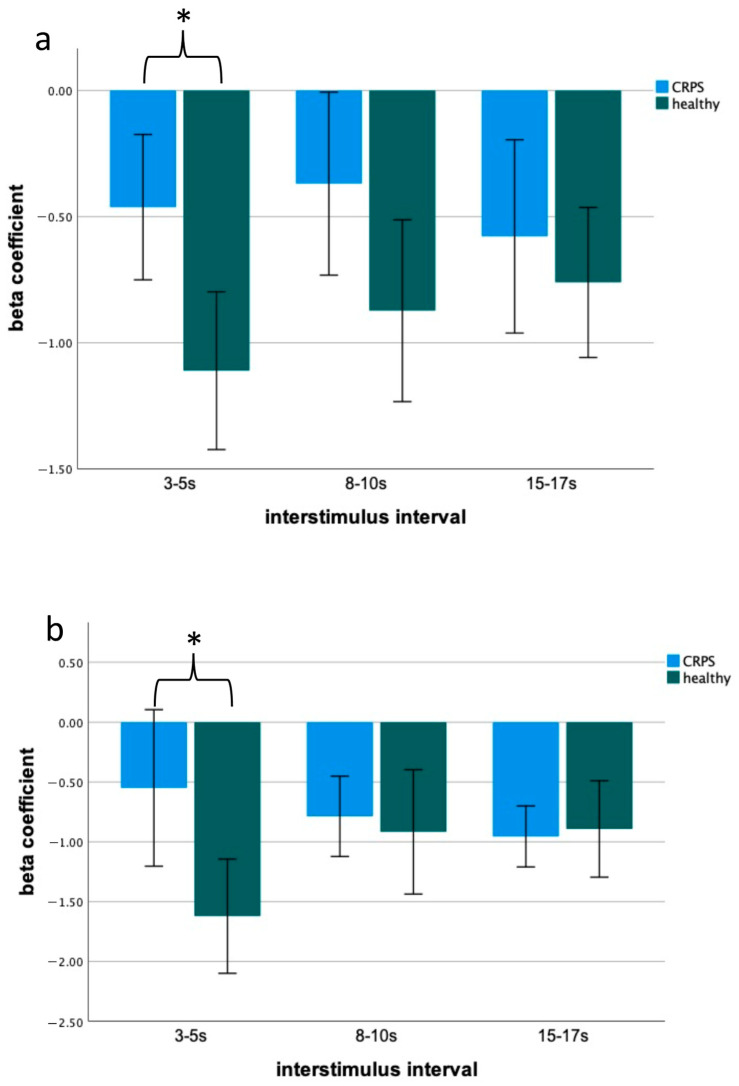
Habituation of nociceptive blink reflex (nBR) for discrete time intervals in patients with CRPS and healthy controls for the stimulated (**a**) and non-stimulated sides (**b**). There was less habituation in the CRPS group for an ISI of 3–5 s (* *p* < 0.05).

There was a significant group (CRPS, HCs) effect in blink reflex habituation (ipsilateral: F (1,25) = 12.91; *p* = 0.001; contralateral: F (1,25) = 4.36; *p* = 0.048). The post hoc analysis showed that habituation only at the short interstimulus interval of 3–5 s showed significant differences between patients and HCs (ipsilateral: t (24) = 3.32; *p* = 0.01; Cohen’s d: 1.3 contralateral: t (24) = 2.88; *p* = 0.04; Cohen’s d: 1.1; see Figure 2).

For the area under R2, there was a group difference between patients with CRPS only for the stimulated side (F (1,25) = 5.52; *p* = 0.027). After multiple corrections, the post hoc test showed a trend for an increased R2 area at all interstimulus intervals (R2: 3–5 s: t (24) = 2.1; *p* = 0.07; 8–10 s: t (24) = 2.2; *p* = 0.06; 15–17 s: t (24) = 2.3; *p* = 0.05).

There were no significant associations between the clinical characteristics (current pain levels, CRPS severity, and CRPS duration) and the electrophysiological parameters (R2a and R2h).

### 2.3. Blink Reflex as a Potential Biomarker for CRPS

**Figure 3:**
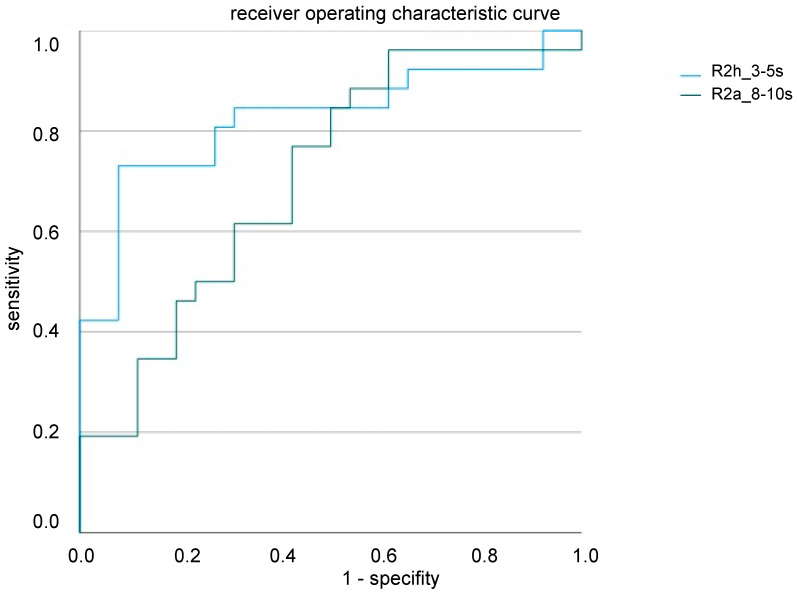
Receiver operating characteristic curve (ROC) analysis revealed a good performance of our prediction model for R2 habituation at an interstimulus interval of 3–5 s (R2h_3–5 s 0.82) and R2 amplitude at an interstimulus interval of 8–10 s (R2a_8–10 s 0.76).

The GLM models (including the stimulation side, R2 habituation, and R2 area of all ISI) revealed habituation of only the R2 component at an ISI of 3–5 s and R2 amplitude at 8–10 s as valid factors for differentiating the CRPS patients and healthy controls (R2h_3–5 s: β = 1.71, *p* = 0.001, R2a_7–9 s: β = 0.12, *p* = 0.024). ROC analysis revealed a good performance of our model (R2h_3–5 s 0.82; R2a_8–10 s 0.76 ROC curve) with good sensitivity and specificity, especially for R2 habituation (Figure 3).

## 3. Discussion

This study provides the first evidence of altered nBR habituation and verifies the findings of altered nBR excitability [19], indicating impaired pain processing at a brainstem level in patients with CRPS. Furthermore, here we propose the habituation of nBR as a valid diagnostic biomarker for CRPS and for central sensitization in general.

### 3.1. Blink Reflex and Brainstem Involvement in CRPS Pathophysiology

Although electrophysiological methods such as transcranial magnetic stimulation [22] or EEG [23] are widely used to investigate maladaptive neuroplasticity in CRPS, studies using the nociceptive blink reflex as an applicable non-invasive electrophysiological method are rare in CRPS. So far, there has been only one study that used this method to investigate the excitability of brainstem interneurons in patients with CRPS. With respect to electrophysiology, Drummond et al. [20] found bilateral reduced R2 amplitudes in CRPS patients compared to HCs, whereas R2 habitation was not investigated.

Here, we used a more comprehensive protocol investigating the blink reflex (R2 area and R2 habituation) in CRPS patients. The result of deficient habituation during stimulus repetition in our CRPS cohort is of high interest since a reduced habituation response of the blink reflex has been reported in patients with migraine [10,24] and is thought to generally reflect impaired information processing between the brainstem, thalamus, and higher cortical areas [25]. The results of impaired nociceptive blink reflex habituation in CRPS, demonstrating disinhibition as a source of increased brainstem excitability to painful stimulation remote from the limb directly affected by CRPS, further support actual pathophysiological concepts in neuropathic pain. Here, impaired central information processing, altered excitability, and mechanisms of disinhibition of brainstem pain-modulation circuits are thought to result in a pro-nociceptive state, contributing to a reduced analgesic ability and an increased sensitivity to incoming somatosensory stimuli [8].

However, we found significantly reduced habituation of R2 at short interstimulus intervals and a trend for a greater R2 area independently from the interstimulus interval. Regarding the method, it is known that the blink reflex can provide information on peripheral and central neurological functions [26] and that the monomorphic first response reflects the monosynaptic excitation of brainstem interneurons, whereas the R2 component reflects a polysynaptic pathway of nociceptive afferences throughout the tractus spinalis trigeminalis to second-order neurons located on the nucleus spinalis trigeminalis [11,27]. From here, a multisynaptic system connects to the ipsilateral and contralateral facial nuclei, including a complex network of the reticular formation [11] (for a detailed overview of brainstem pathways, see Figure 1). This is notable since it is known that the reticular formation as part of the tegmentum contains centers (e.g., periaqueductal grey, raphe nuclei) that belong to the transmission and modulation of nociceptive information, including areas that modulate the extent of pain response and thus could also be a key structure in the pathophysiology of CRPS [12,25]. For the R2 component, it has been demonstrated that neuronal response habituates after repetitive stimulation, especially when using short interstimulus intervals. While the R2 component habituates readily in healthy people, patients with persistent facial [19] pain or migraine [10] showed a substantial reduction in habituation. Therefore, our results fit the existing literature on other diseases involving brainstem pathways in pathophysiology.

The extent to which the alterations in brainstem processing depend on the stage and phenotype of CRPS remains unclear since clinical aspects and electrophysiological parameters were not associated in our cohort. Here, investigating the nBR in larger cohorts of CRPS patients in different disease stages and different clinical phenotypes (predominantly central vs. peripheral phenotype) would be necessary to investigate the role of the brainstem in the development and chronification of CRPS.

### 3.2. Blink Reflex as a Potential Biomarker of CRPS

In our actual cohort of patients, using a generalized linear model revealed that the habituation of R2 at an interstimulus interval of 3–5 s and the R2 area at 8–10 s had the potential to discriminate between the CRPS and healthy groups. These results generate the first evidence for the blink reflex to have high sensitivity and specificity as a potential diagnostic biomarker by the definition of the FDA Biomarker Working Group [28]. Since alterations of habituation have been reported in other pain diseases [10,19,29], it must be hypothesized here that they are not disease-specific, but rather indicate impaired sensory processing at the brainstem level in general. Based on this, the blink reflex may be considered a diagnostic biomarker for various pain conditions pathophysiologically involving mechanisms of central sensitization. Knowledge of the potential to discriminate between different neuropathic pain conditions and CRPS would further increase the significance of the nBR, especially as the clinical diagnostic criteria showed good sensitivity but only moderate specificity [30].

Future (longitudinal) investigations using this easy-to-perform, noninvasive, and highly reproducible method are necessary to assess the potential of the nBR in monitoring treatment response (therapeutic biomarker), as has recently been demonstrated in patients with migraine [10].

A perspective identifying a valid diagnostic and treatment biomarker will be increasingly important since there is evidence that long times between onset and diagnosis and delayed (insufficient) therapy are predictive for CRPS chronification [31], which is associated with persistent severe pain, impaired sensorimotor function, and reduced quality of life.

### 3.3. Limitations

The interpretation of the current results should consider a few limitations. First, our small sample size did not allow us to investigate alterations of the nBR in the context of different phenotypes of CRPS. Including larger cohorts of patients with either predominantly peripheral or central phenotypes would help to further understand the role of brainstem alterations and individual patterns of symptoms. Secondly, since we investigated only patients at a chronic stage of the disease, future studies that investigate any variant of the blink reflex as a clinical biomarker should also include patients in the acute and subacute stages to assess its role in disease chronification.

Furthermore, we did not control for patients’ medication in this explorative study, so another limitation is that we cannot exclude the potential pharmacological effects on central disease activity, although modulation of the nBR has only been described for diazepam, which was not taken by our patients [32].

Finally, since sensory disturbance with aspects of hemilateral hyperalgesia predominantly appeared on the affected side and electrical stimuli were delivered above the pain threshold, it would be interesting to assess the impact of the stimulation side, pain threshold, and different forms of nociceptive stimulation (e.g., heat pulses or laser stimuli) in further studies [20].

## 4. Materials and Methods

### 4.1. Participants

Patients were recruited through support groups in Northern Germany. The inclusion criteria were an age between 18 and 85 years and unilateral chronic CRPS type II of the upper limb. The exclusion criteria were the affection of other limbs, neurological (including primary headache syndromes) or psychiatric diagnoses other than CRPS, and other chronic pain syndromes.

Age and gender-matched healthy volunteers served as controls (HCs), with the exclusion criteria of chronic pain or neurological or psychiatric disease.

All participants gave their written informed consent. The study was approved by the local ethics committee (BB 067/21). The study design, recruitment, and endpoints were prospectively registered in the German Clinical Trials Register (DRKS0002661).

### 4.2. Assessment of Demographics and Clinical Characteristics

The general demographic characteristics, including age and gender, were recorded for all participants. The presence of CRPS was rechecked according to the current diagnostic criteria [33]. In addition, CRPS-specific characteristics, including an initial event, side of the affected limb, and disease duration, as well as comorbidities and actual medication, were assessed at study inclusion (see Table 1 for an overview of the demographic and clinical data of the 13 CRPS patients).

Pain levels at rest and during movement (clenching and unclenching the affected fist 5 times) on the day of the examination were assessed using a 10 cm visual analog rating scale, with 0 indicating no pain and 10 indicating the worst imaginable pain.

Disease severity was classified using the CRPS severity score (range: 0–17, higher numbers indicate worse CRPS) [30].

### 4.3. Assessment of Nociceptive Blink Reflex Habituation and Sensitization

**Figure 4:**
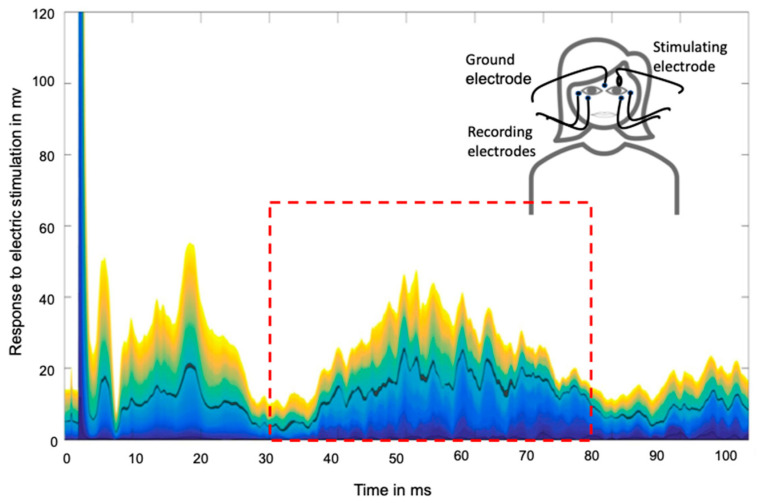
Three superimposed 20 nBR traces (represented by different colors) of one participant and schematic demonstration of methodological setting. Black line: calculated mean of the 20 nBR traces with an interstimulus interval of 3–5 s. The dotted red box indicates the time window of the R2 component.

The stimulation and data recording were performed with a commercial electrophysiology setup (Neuropack X1, Nihon Kohden Europe, Rosbach, Germany) using a bipolar montage of gold cup electrodes, which were fixed using adhesive paste and tape. Electrical stimulation was applied to the supraorbital division of the trigeminal nerve on the affected hand side (matched in controls). Blink reflex responses were recorded at the stimulated and non-stimulated sides (for details, see Figure 4).

The pain threshold (PT) was established using electrical stimuli, which were at least 30 s apart to avoid habituation, at increasing intensity. Then, 60 stimuli with a pulse width of 0.3 ms and 1.5 × PT intensity were applied in 6 blocks consisting of 10 stimuli each. It is known that habituation of the blink reflex can be elicited at different interstimulus intervals with shorter intervals, causing more habituation [24]. Since most studies have used intervals between 3 s and 17 s [29,34,35] and systematic investigations of the impact of different ISI in different pain conditions are lacking, we delivered the stimuli with three different interstimulus intervals (ISI; 3–5 s, 8–10 s, and 15–17 s). Each ISI was stimulated twice in a pseudorandomized order with an interblock interval of at least 2 min to avoid habituation effects [10,16].

Traces of the recorded EMG responses were then exported into a MATLAB environment, and the R2 component was defined as responses that occurred in an interval of 30–80 ms (see Figure 4).

### 4.4. Data Evaluation

The NBR data were pre-processed as described following the algorithm proposed by Thiele et al. [10]. The area of R2 responses (R2a) was computed using a trapezoidal approximation of its integral. Habituation (R2h) was quantified as the beta coefficient (i.e., β0, slope) of the linear regression: f(R2ai) = β0 × 2ai + intercept (I = stimulus order), whereas a positive slope indicates facilitation, a negative slope indicates habituation, and zero slope indicates no change in the trigemino-cervical complex (TCC) to consecutive stimulation.

### 4.5. Sample Size Estimation and Statistical Analysis

Sample size estimates were based on the effect sizes observed in studies of migraine pathophysiology [34,35]. These found that habituation deficits of the NBR can be expected with a mean effect size of 1.3 (Cohen’s d) when compared to healthy controls. G*Power (v.3.1.9.2, University of Düsseldorf, Düsseldorf, Germany) was used to estimate the sample size required to investigate the hypothesis that habituation of R2 changes such as those observed in migraine can be found in patients with CRPS as compared to healthy controls. The primary hypothesis was to be tested using a one-sided t-test with an alpha-error of 0.05 and power of 90%, which requires a sample size of 11 per group.

Further statistical analyses were carried out using the Statistical Package for the Social Sciences (SPSS v25.0, IBM, Armonk, NY, USA.) and MATLAB (R2018a, The Mathworks. Natick, MA, USA).

The primary hypothesis was assessed using a repeated-measures ANOVA (rmANOVA) considering the different interstimulus intervals (3–5 s, 8–10 s, and 15–17 s) as within-subject factors and groups (CRPS, healthy) as between-subject factors. The post hoc independent t-tests described above were used for discrete time intervals. Alpha inflation was corrected for using the Bonferroni method (three different interstimulus intervals; stimulated and non-stimulated side). Secondary analyses aimed to investigate the subset of R2 parameters which best separated the CRPS cases from the controls. For this purpose, we used a generalized linear model (GLM; logit link function, including possible interactions) and an automated stepwise optimization of the Akaike information criterion, which led to the identification of the least sufficient subset of predictors (factor: stimulation side; covariates: habituation, excitability) for the data (i.e., binary group assignment). The GLM optimization stopped when the AIC could not be improved by adding or removing any predictor or interaction.

Age and gender were not considered to be valid predictors since patients with CPRS and healthy participants that were recruited were age- and gender-matched. For significant predictors, a receiver operating characteristic curve (ROC) was used to evaluate the performance in discriminating between the groups.

Since clinical data (CRPS duration, pain levels) were not normally distributed (Shapiro–Wilk), Spearman correlation coefficients were determined to evaluate whether the electrophysiological parameters correlated with the clinical data.

## 5. Conclusions

This study provides the first evidence of altered nBR habituation in chronic CRPS and verifies the findings of altered nBR excitability, indicating impaired pain processing at a brainstem level in those patients. The results consider the polysynaptic R2 response of the nociceptive blink reflex to be a diagnostic biomarker for CRPS. Future studies should evaluate the blink reflex as a clinical biomarker to predict treatment response or disease progression of CRPS.

## Figures and Tables

**Table 1:** Overview of demographic and clinical data of 13 CRPS patients.

Patient No	Age	Gender ^1^	Affected Limb ^2^	Inciting Event ^3^	Time Onset ^4^	Rpain ^5^	Mpain ^6^	CSS ^7^	Medication ^8^
1	22	f	R	O	58	6	7	15	ABCD
2	67	f	R	CT	120	5	6	13	A
3	50	m	R	WF	144	6	7	15	ABD
4	54	f	L	CT	132	5	6	13	CD
5	69	m	L	WF	84	2	2	7	
6	66	f	L	O	50	5	6	10	ABD
7	49	m	L	O	96	6	9	15	ABCD
8	82	f	R	WF	36	2	9	7	
9	21	f	R	AR	84	6	10	14	BD
10	66	m	R	WF	60	3	5	8	ACD
11	49	f	L	RF	36	2	10	9	AC
12	61	f	R	CT	96	7	8	10	BCD
13	43	f	R	RF	12	7	10	11	ABCD

Note: ^1^ f: female. m: male; ^2^ L: left. R: right; ^3^ RF: radius fracture; CT: carpal tunnel surgical intervention; O: other surgery; WF: wrist fracture; AR: allergic reaction; ^4^ time from CRPS onset in months; ^5^ Rpain: rest pain) (10 = max pain); ^6^ Mpain: movement pain () (10 = max pain); ^7^ CSS: CRPS severity score at baseline; ^8^ A: non-steroidal anti-inflammatory drugs (NSAIDs); B: opioid; C: antidepressants; D: anticonvulsants.

## Data Availability

The data presented in this study are available upon request from the corresponding author.
